# Involvement of oxidative stress in protocatechuic acid‐mediated bacterial lethality

**DOI:** 10.1002/mbo3.472

**Published:** 2017-03-27

**Authors:** Taofeek O. Ajiboye, Ramat S. Habibu, Kabiru Saidu, Fatimah Z. Haliru, Hikmat O. Ajiboye, Najeeb O. Aliyu, Oluwayemisi B. Ibitoye, Judith N. Uwazie, Hamdalat F. Muritala, Sharafa A. Bello, Idris I. Yusuf, Aisha O. Mohammed

**Affiliations:** ^1^ Antioxidants, Redox Biology and Toxicology Research Laboratory Department of Biological Sciences Al‐Hikmah University Ilorin Nigeria; ^2^ Department of Biochemistry University of Ilorin Ilorin Nigeria; ^3^ Microbiology Unit Department of Biological Sciences Al‐Hikmah University Ilorin Nigeria

**Keywords:** Antioxidant enzymes, bacteria, electron transport chain complex, fragmented DNA, hydroxyl radical, NAD^+^/NADH, protocatechuic acid, superoxide anion radical

## Abstract

The involvement of oxidative stress in protocatechuic acid‐mediated bacterial lethality was investigated. Minimum inhibitory concentrations (MIC) and minimum bactericidal concentration (MBC) of protocatechuic acid against *Escherichia coli*,* Pseudomonas aeruginosa*, and *Staphylococcus aureus* are 600 and 700 μg/ml, 600 and 800 μg/ml, and 600 and 800 μg/ml, respectively. The optical densities and colony‐forming units of protocatechuic acid‐treated bacteria decreased in time‐dependent manner. Protocatechuic acid (4× MIC) significantly increased the superoxide anion content of *E. coli*,* P. aeruginosa*, and *S. aureus* compared to dimethyl sulfoxide (DMSO). Superoxide dismutase, catalase, and NAD^+^/NADH in protocatechuic acid‐treated *E. coli*,* P. aeruginosa*, and *S. aureus* increased significantly when compared to DMSO. Conversely, level of reduced glutathione decreased in protocatechuic acid‐treated *E. coli*,* P. aeruginosa*, and *S. aureus*, while glutathione disulfide increased when compared to DMSO. Furthermore, malondialdehyde and fragmented DNA increased significantly following exposure to protocatechuic acid. Protocatechuic acid inhibited the activity of complexes I and II. From the above findings, protocatechuic acid enhanced the generation of reactive oxygen species (superoxide anion radical and hydroxyl radical) in *E. coli*,* P. aeruginosa*, and *S. aureus*, possibly by autoxidation, fenton chemistry, and inhibiting electron transport chain resulting in lipid peroxidation and DNA fragmentation and consequentially bacterial cell death.

## 1 Introduction

Reactive oxygen species, such as superoxide anion radical, hydrogen peroxide, and hydroxyl radical, are inevitable by‐products of aerobic respiration (Storz & Imlay, 1999). These oxygen‐derived species possess potent antimicrobial activity (Dixon & Stockwell, 2014; Fang, 2011), which largely results from perturbation of antioxidant and prooxidant balance in favor of latter. Redox perturbation in bacteria occurs from both exogenously and endogenously generated ROS that overwhelm bacterial antioxidant defense system. While environmental chemicals with prooxidant potentials form exogenous source of ROS, mitochondrial electron transport chain is the chief source of endogenously generated ROS (Nickel, Kohlhaas, & Maack, 2014). The primary targets of these ROS are cellular protein, lipids, and DNA.

Recent studies have documented the involvement of ROS in bacterial lethality of antibiotics and antimicrobials (Ajiboye et al., 2016a,b,c,d; Kohanski, Dwyer, Hayete, Lawrence, & Collins, 2007). In these studies, hydroxyl radical, generated through the catalytic action of ferrous ion on hydrogen peroxide (Fenton chemistry) (Lemire, Harrison, & Turner, 2013), is responsible for bacterial death. Plant derived phytochemicals, including phenolics with catechol ring, possess prooxidant activity (Schweigert, Zehnder, & Eggen, 2001). However, no studies have reported any relationship between their capability to generate ROS and bacterial lethality.

Protocatechuic acid (3,4‐dihydroxybenzoic acid) is a benzoic acid derivative found in vegetables, nuts, brown rice, fruits, and herbal medicines (Da‐Costa‐Rocha, Bonnlaender, Sievers, Pischel, & Heinrich, 2014). Studies have demonstrated the anticarcinogenic, antioxidants, cytotoxic, free radical scavenging, apoptotic, and cell cycle arrest activities (Ferreira, Barros, & Abreu, 2009; Yin, Lin, Wu, Tsao, & Hsu, 2009; Yip, Chan, Pang, Tam, & Wong, 2006). Experimental findings have also demonstrated the prooxidant activity of protocatechuic acid in cell‐free and in vitro cellular systems (Simić, Manojlović, Šegan, & Todorović, 2007; Zeraik et al., 2014). In this study, we demonstrated that protocatechuic acid promotes redox‐related biochemical changes leading to death of *Escherichia coli*,* Pseudomonas aeruginosa*, and *Staphylococcus* aureus.

## 2 Materials and Methods

### 2.1 Bacteria strains

*Escherichia coli* (ATCC 25922), *Pseudomonas aeruginosa* (ATCC 27853), and *Staphylococcus aureus* (ATCC 29213) were procured from American Type Culture Collection and propagated on a Luria–Bertani (LB) at 37°C.

### 2.2 Chemicals

Dimethyl sulfoxide, diphenylamine, 5,5′‐dithiobis(2‐nitrobenzoic acid), epinephrine, guanidine hydrochloride, hydrogen peroxide, N‐ethylmaleimide, sodium chloride, thiobarbituric acid were procured from Sigma‐Aldrich (St. Louis, MO). Nitroblue tetrazolium, 2,2′‐dipyridyl, l‐glutathione (reduced), and protocatechuic acid are products of Santa Cruz Biotechnology. All other reagents are products of Sigma‐Aldrich (St. Louis, MO).

### 2.3 Minimum inhibitory concentration and minimum bactericidal concentration

Minimum inhibitory concentration (MIC) of protocatechuic acid was determined using 96‐well microtiter plate as described by Balouiri, Sadiki, and Ibnsouda (2016). Inocula (10^4^ CFU/ml) were mixed with protocatechuic acid in 96‐well microtiter plate to give concentrations (1–10^9^ ng/ml). The culture medium containing 0.04% DMSO in sterile distilled water served as control. Plates were incubated for 24 hr at 37°C. MIC was expressed as the lowest concentration which inhibited growth, judged by lack of turbidity in the well. The turbidity in each well was then determined using Versamax microplate reader (Molecular Devices, CA, USA). The experiment was performed in triplicate and repeated three times along with reference antibiotics, ciprofloxacin. The content of wells lacking turbidity were aspirated into eppendorf tubes, and centrifuged to collect the cells. The cells were washed to aseptically remove protocatechuic acid, diluted with 0.9% NaCl, mixed with molten soft LB agar (0.8%) at 42°C, and poured onto agar plates containing solid LB agar (1.5%). Colonies were counted after 24 hr at 37°C. The concentration that completely inhibited colony formation, despite the treatment with test compound for 48 hr, was aseptically removed and transferred into a new agar plate to examine survival at 24 hr.

### 2.4 Time–kill bacterial susceptibility assay

Susceptibility of *E. coli*,* P. aeruginosa*, and *S. aureus* to protocatechuic acid was investigated using the procedure described by Ajiboye et al. (2016b). Briefly, organisms were grown overnight in LB medium, harvested by centrifugation, and resuspended in 50 ml fresh medium (LB) to OD_600_ = 0.1, and grown aerobically at 37°C in 250 ml flask to OD_600_^ ^= 0.2. Protocatechuic acid was added to the culture to obtain concentration (4× MIC) or dimethyl sulfoxide (DMSO) and incubated at 37°C for 3 hr. Absorbance of the incubation medium was read at 600 nm for every 30 min interval of 3 hr incubation time. For colony formation, samples of control culture (DMSO‐treated culture) and cultures treated with protocatechuic acid (4× MIC) were removed at intervals (0, 30, 60, 90, 120, 150, and 180 min) and centrifuged to collect the cells as pellet. The cells were washed and diluted with 0.9% NaCl, mixed with molten soft LB agar (0.8%) at 42°C, and poured onto agar plates containing solid LB agar (1.5%). Colonies were counted after 24 hr at 37°C.

### 2.5 Preparation of cell‐free extract

Cell‐free extract was prepared from the samples obtained after 3 hr incubation of organisms with protocatechuic acid (4× MIC). Cells were harvested by centrifugation, washed twice, and suspended in sucrose‐Tris buffer (25 mmol/L sucrose solution, 10 mmol/L Tris‐HCl, pH 7.4). Glass beads (2 g) were added to the bacterial suspension, homogenized, and centrifuged at 3,000 *g* for 10 min at 4°C to obtain the cell‐free extract as the supernatant.

### 2.6 Oxidative stress biomarkers

#### 2.6.1 Superoxide dismutase

The activity of superoxide dismutase (SOD) was determined according to Misra and Fridovich (1972). Briefly, 10 μl of cell‐free extract was added to 125 μl of 0.05 mol/L carbonate buffer (pH 10.2) to equilibrate, and the reaction was started by addition of 15 μl of freshly prepared 0.3 mmol/L epinephrine. The increase in absorbance at 480 nm was recorded every 30 s for 150 s using Versamax microplate reader (Molecular Devices). One unit of enzyme activity was defined as 50% inhibition of the rate of autoxidation of epinephrine as determined by change in absorbance min^−1^ at 480 nm.

#### 2.6.2 Catalase

The cell‐free extract was evaluated for catalase activity using the procedure described by Chen, Liu, Zhu, Xu, and Li (2010). Briefly, cell‐free extract (10 μl) was mixed thoroughly with cold 6 mmol/L H_2_O_2_ (100 μl). The reaction was stopped by mixing with 3 mol/L H_2_SO_4_ (20 μl) followed by 0.01 KMnO_4_. Reaction mixture was vortexed and absorbance read at 480 nm within 30–60 s using Versamax microplate reader (Molecular Devices).

#### 2.6.3 Reduced glutathione (GSH) and glutathione disulfide (GSSG)

The level of GSH in the cell‐free extract was determined using the procedure described by Ellman (1959). Cell‐free extract (20 μl) was mixed with 170 μl of 0.1 mol/L potassium phosphate buffer (pH 7.4). The reaction was started by adding 10 mmol/L DTNB (10 μl) and incubated for 30 min at room temperature. Absorbance of the reaction mixtures was read at 412 nm using Versamax microplate reader (Molecular Devices).

GSSG level was determined using the procedure described by Hissin and Hilf (1976). Cell‐free extract (50 μl) was mixed with 20 μl of 0.04 mol/L *N‐*ethylmaleimide (NEM) to prevent oxidation of GSH to GSSG. It was incubated at room temperature for 30 min and 1.68 ml of 0.3 mol/L Na_2_HPO_4_ solution was added to it followed by 250 μl of DTNB reagent. The absorbance of the sample was measured at 412 nm.

#### 2.6.4 NAD^+^/NADH

The NAD^+^/NADH ratio of bacteria cells was assessed using the Sigma‐Aldrich assay kit (MAK037). Cells were washed with cold phosphate‐buffered saline and centrifuged at 2,000*g* for 5 min. Cell was extracted with 400 μl of NAD^+^/NADH extraction buffer by homogenization or freeze/thawing for two cycles of 20 min on dry ice followed by 10 min at room temperature. To remove insoluble material, the samples were vortexed for 10 sec and then centrifuged at 13,000*g* for 10 min. Extracted NAD^+^/NADH supernatant was transferred into a labeled tube. The supernatant was then used for NAD^+^/NADH assay.

#### 2.6.5 Malondialdehyde

Malondialdehyde content of cell‐free extract was determined as described by Reilly and Aust (2001). Briefly, cell‐free extract was mixed with TBA/TCA/HCl (15%, 0.37%, 0.2 N) at a reagent/sample ratio of 2:1 (v/v), placed in a boiling water bath for 15 min, cooled to room temperature, and centrifuged at 1,000 g for 10 min at room temperature. The absorbance of the solution was read at 535 nm against the blank (containing all reagents except hepatocytes suspension) using Versamax microplate reader (Molecular Devices). MDA content was determined using the extinction coefficient of 1.56 × 10^6^.

#### 2.6.6 Fragmented DNA

The percentage fragmented DNA in the cell‐free extract was determined using the procedure described by Burton (1956). Briefly, cell‐free extract was centrifuged at 15,000*g* for 15 min at 4°C. The supernatant was separated from the pellet and treated with trichloroacetic acid (1.50 ml, 10%). The pellet was also treated with trichloroacetic acid (0.65 ml, 5%). The reaction mixtures were allowed to precipitate overnight (≥4 hr) in a refrigerator (4°C), and centrifuged at 2,500 *g* for 10 min. The reaction mixtures were boiled at 100°C for 15 min, cooled to room temperature, and centrifuged at 2,500 *g* for 5 min. The supernatants (50 μl) were treated with diphenylamine reagent (100 μl) and incubated at 37°C for 4 hr. Absorbance was read at 600 nm using Versamax microplate reader (Molecular Devices). The fragmented DNA was calculated using the following expression:

$$\text{Fragmented DNA}\left(\%\right)=\frac{\text{Absorbance of the supernatant}}{\text{Absorbance of the supernatant + Absorbance of pellet}}$$

#### 2.6.7 Electron transport chain complexes

The activities of NADH:ubiquinone oxidoreductase (complex I; EC 1.6.5.3) and succinate:ubiquinone oxidoreductase (complex II; EC 1.3.5.1) in the cell‐free extract of *E. coli*,* P. aeruginosa*, and *S. aureus* were assayed using the procedures described by Van Bergen, Blake, Crowston, and Trounce (2014). Activities of NADH:ubiquinone oxidoreductase and succinate:ubiquinone oxidoreductase were calculated using extinction coefficients 6.22 and 19.1 mmol/L^−1^_ _cm^−1^, respectively.

### 2.7 Involvement of reactive oxygen species (superoxide anion radical and hydroxyl radical) in bacterial lethality

#### 2.7.1 Superoxide anion radical

For superoxide anion radical generation, cells (1 ml) in exponential phase were incubated with protocatechuic acid for 30 min, followed by adding nitroblue tetrazolium (0.5 ml, 1 mg/ml) and incubated for 30 min at 37°C. After incubation, 0.1 ml of HCl (0.1 mol/L) was added and centrifuged at 1,500*g* for 10 min. The reduced nibtroblue tetrazolium in the pellets was extracted with DMSO, diluted with 0.8 ml phosphate‐buffered saline (pH 7.5) and the absorbance was read at 575 nm using Versamax microplate reader (Molecular Devices). The superoxide anion of the cells was calculated using the molar extinction coefficient of 3‐(4,5‐dimethylthiazol‐2‐yl)‐2,5‐diphenyltetrazolium bromide formazan (17,000 M^−1 ^cm^−1^ at pH 7.4–8.0) as described by Ajiboye et al. (2016b).

#### 2.7.2 Hydroxyl radical

In this assay, *E. coli*,* P. aeruginosa*, and *S. aureus* were grown overnight in LB medium, harvested by centrifugation and resuspended in 50 ml fresh medium (LB) to OD_600_ = 0.1, and grown aerobically at 37°C in 250 ml flask. At mid‐log phase (OD_600_ = 0.5), 2,2′‐dipyridyl (500 μmol/L), thiourea (150 mmol/L), and/or protocatechuic acid (4× MIC) were added and incubated at 37°C for 3 hr. Absorbance of the incubation medium was read at 600 nm for every 20 min interval of 3 hr incubation time using Versamax microplate reader (Molecular Devices). In addition, samples of control culture and treated culture were removed at intervals of 0, 20, 40, 60, 80, 100, 120, 140, 160, and 180 min and centrifuged to collect the cells as pellet. The cells were washed and diluted with 0.9% NaCl, mixed with molten soft LB agar (0.8%) at 42°C, and poured onto agar plates containing solid LB agar (1.5%). Colonies were counted using digital colony counter after 24 hr at 37°C.

### 2.8 Statistical analysis

Results were expressed as the mean of three independent experiments ± standard deviation. One‐way analysis of variance (ANOVA) followed by Student's *t*‐test was used to detect any significant difference (*p *<* *.05) between the treatments using Statplus, 2011 (AnalystSoft, Inc., Alexandria, VA, USA).

## 3 Results

### 3.1 Minimum inhibitory concentration and minimum bactericidal concentration

MIC and MBC are widely used in determining the potency of antimicrobial agents. We determined the MIC and MBC values of protocatechuic acid against *E. coli*,* P. aeruginosa*, and *S. aureus* (Table 1). The MIC and MBC values of protocatechuic acid are relatively higher than that of synthetic reference antibiotic, ciprofloxacin. This is not surprising as a lower MIC and MBC could pose threat to human, since this compound is widely available in fruits at higher concentrations.

**Table 1:** Minimum inhibitory concentration (MIC) and minimum bactericidal concentration (MBC) of protocatechuic acid

	Protocatechuic acid (μg/ml)		Ciprofloxacin (μg/ml)	
	MIC	MBC	MIC	MBC
*Escherichia coli* ATCC 25922	550	600	18	64
*Pseudomonas aeruginosa* ATCC 27853	300	400	36	64
*Staphylococcus aureus* ATCC 29213	450	500	36	64

### 3.2 Time–kill bacterial susceptibility

**Figure 1:**
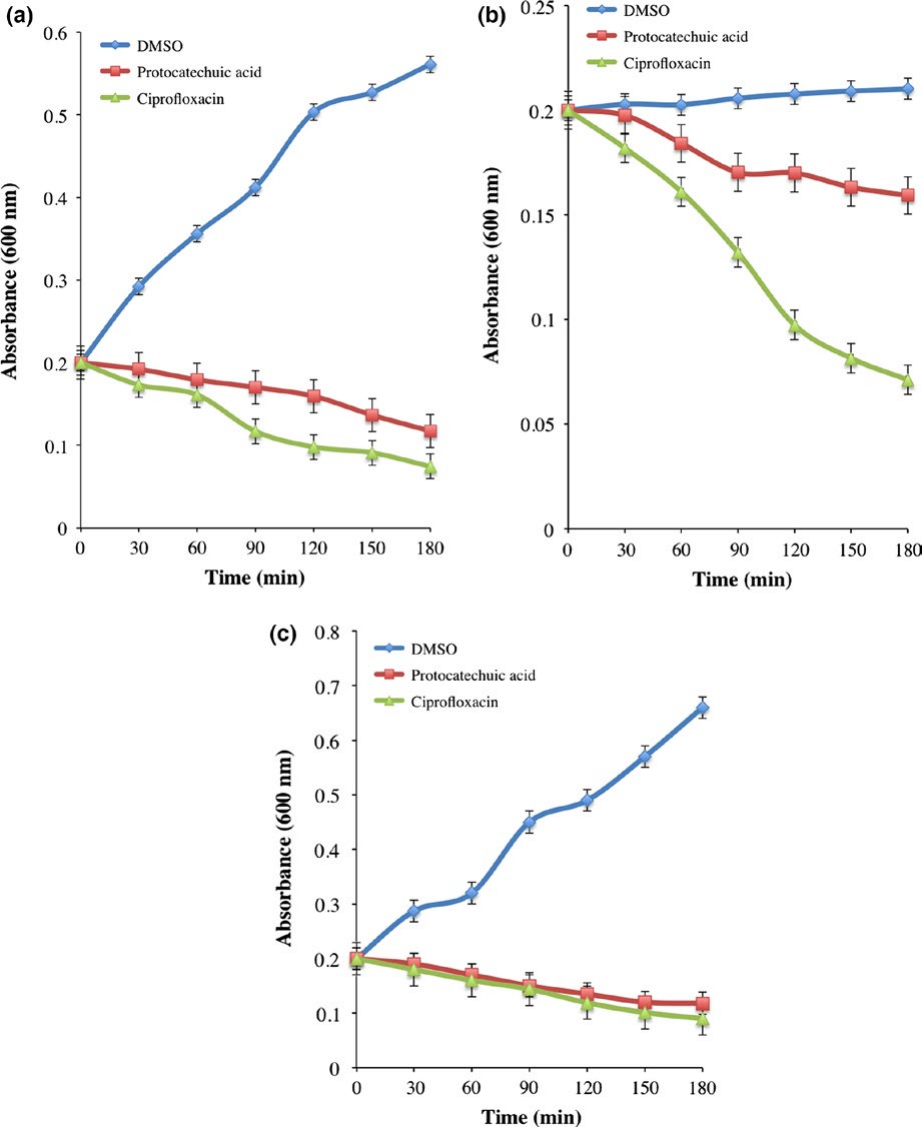
Viability of (a) *Escherichia coli* (ATCC 25922), (b) *Pseudomonas aeruginosa* (ATCC 27853), and (c) *Staphylococcus aureus* (ATCC 29213) exposed to protocatechuic acid (4× MIC). Values are mean ± SEM of three determinations and are statistically significant at *p *<* *.05

**Figure 2:**
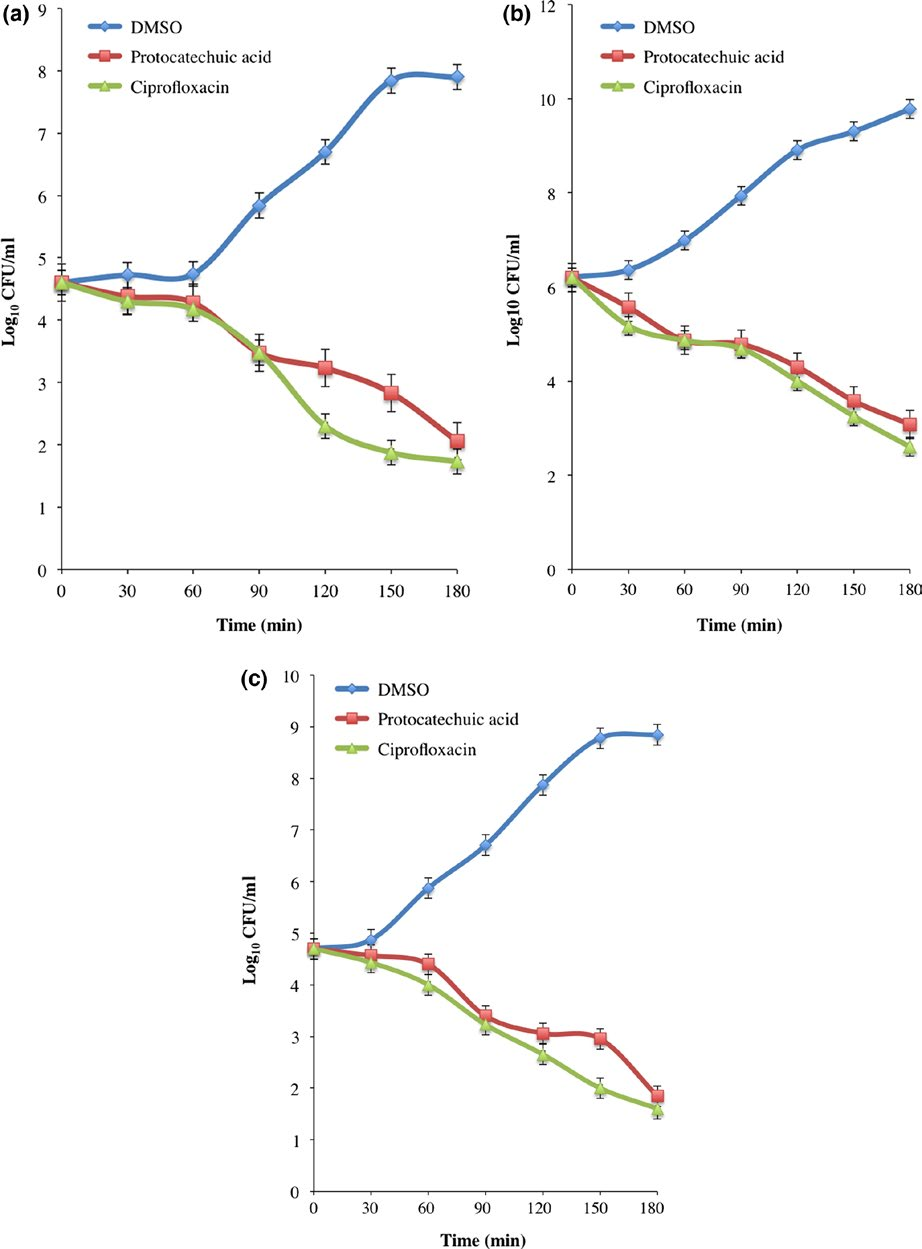
Viability (CFU/ml) of (a) *Escherichia coli* (ATCC 25922), (b) *Pseudomonas aeruginosa* (ATCC 27853), and (c) *Staphylococcus aureus* (ATCC 29213) exposed to protocatechuic acid (4× MIC). Values are mean ± SEM of three determinations and are statistically significant at *p *<* *.05

The change in absorbance of protocatechuic acid (4× MIC)‐treated *E. coli*,* P. aeruginosa*, and *S. aureus* decreased significantly in time‐dependent manner when compared with DMSO‐treated bacteria (Figure 1a). The decrease was more pronounced at 180 min and compared significantly with ciprofloxacin, a reference antibiotic (Figure 1a–c). Furthermore, the decrease in absorbance was supported by reduction in CFU/ml of the organisms following incubation with the protocatechuic acid (Figure 2a–c).

### 3.3 Oxidative stress biomarkers

**Figure 3:**
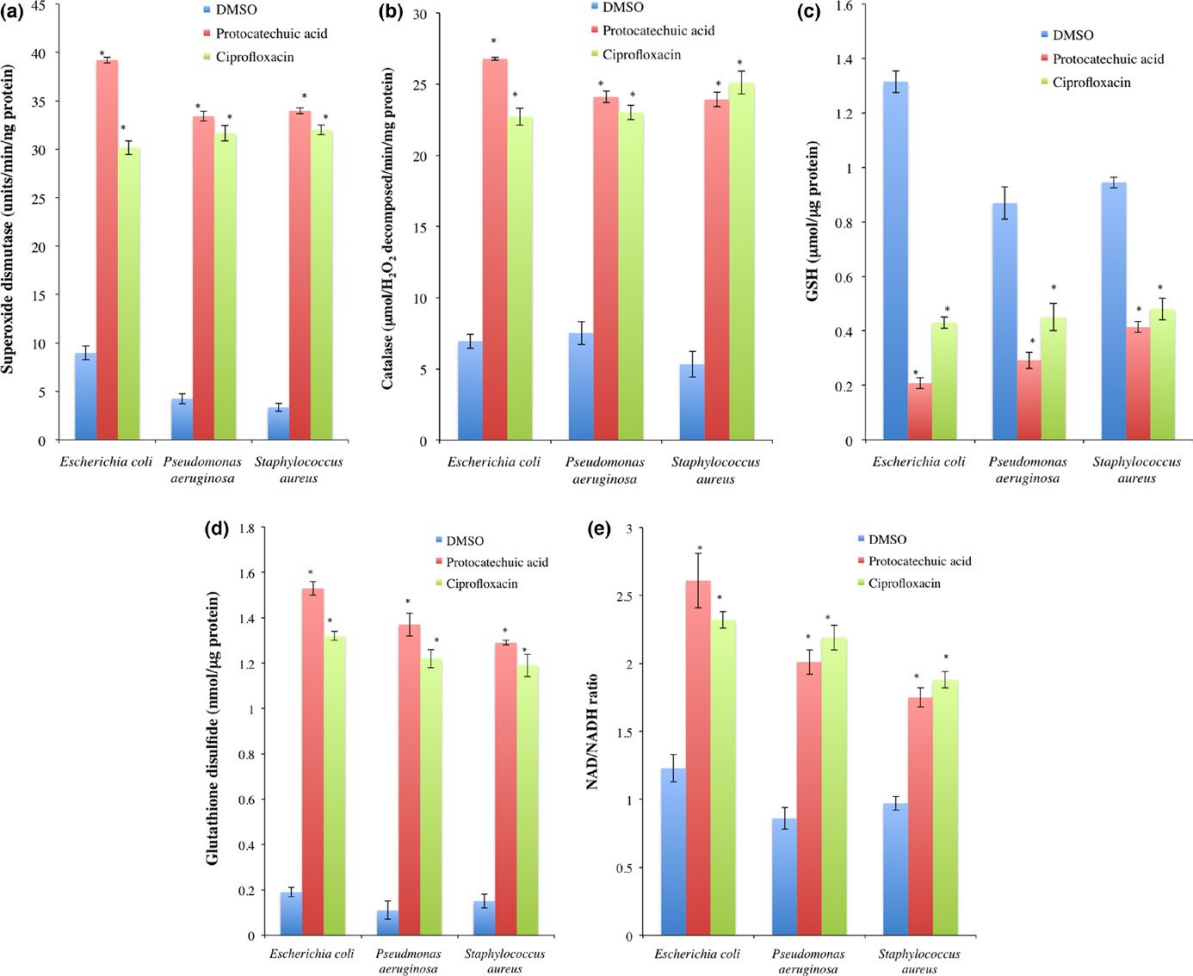
(a) Superoxide dismutase, (b) catalase, (c) reduced glutathione, (d) glutathione disulfide, and (e) NAD ^+^/NADH of *Escherichia coli* (ATCC 25922), *Pseudomonas aeruginosa* (ATCC 27853), and *Staphylococcus aureus* (ATCC 29213) exposed to protocatechuic acid (4× MIC). Values are mean ± SEM of three determinations and are statistically significant at *p *<* *.05. *p <* *.05 vs DMSO. DMSO, dimethyl sulfoxide

The activities of superoxide dismutase and catalase increase (*p *<* *.05) in the cell‐free extracts of protocatechuic acid (4× MIC)‐treated *E. coli*,* P. aeruginosa*, and *S. aureus* when compared to DMSO‐treated organisms (Figure 3a–b). The increased activity of superoxide dismutase was 4.37‐, 7.90‐, and 10.11‐fold when compared to DMSO‐treated cells, while catalase activity increased by 3.85‐, 3.21‐, and 4.50‐fold.

The level of GSH in *E. coli*,* P. aeruginosa*, and *S. aureus* exposed to protocatechuic acid (4× MIC) decreased significantly (*p *<* *.05) by 84.16%, 66.39%, and 56.32%, respectively (Figure 3c). Conversely, GSSG increased significantly in the cell‐free extracts of protocatechuic acid‐treated organisms. This increase was compared significantly with ciprofloxacin‐treated organisms (Figure 3d). In addition, the NAD^+^/NADH ratio of cells exposed to protocatechuic acid increased by >1.5‐fold, indicating increased electron transport chain activity, which was compared with ciprofloxacin‐treated cells (Figure 3e).

**Figure 4:**
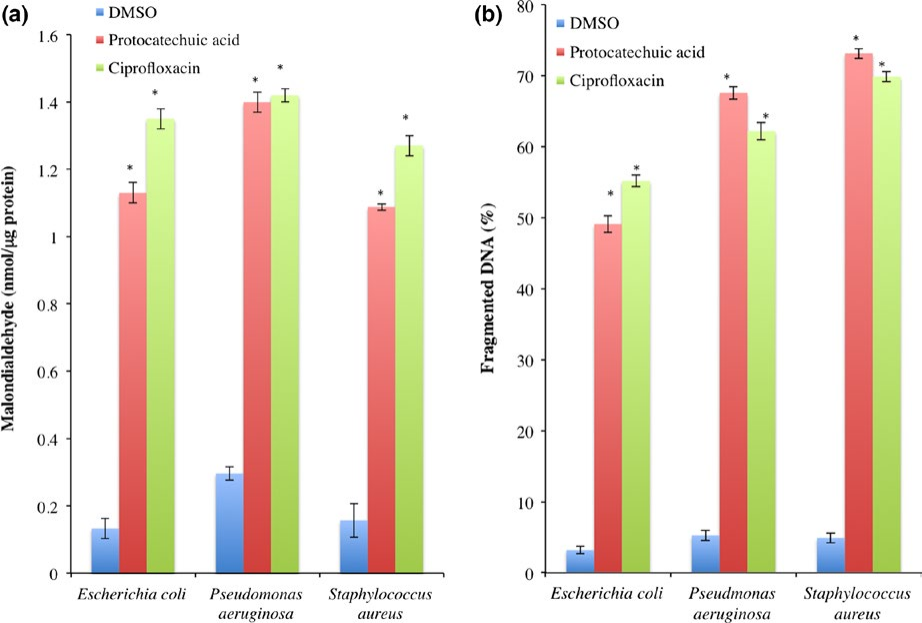
(a) Malondialdehyde and (b) fragmented DNA in protocatechuic acid‐treated *Escherichia coli* (ATCC 25922), *Pseudomonas aeruginosa* (ATCC 27853), and *Staphylococcus aureus* (ATCC 29213). Values are mean ± SEM of three determinations and are statistically significant at *p *<* *.05. *p <* *.05 vs DMSO. DMSO, dimethyl sulfoxide

Malondialdehyde, a product of lipid peroxidation, increased significantly (*p *<* *.05) by 8.56‐, 4.73‐, and 6.95‐fold, respectively, in the cell‐free extracts of *E. coli*,* P. aeruginosa*, and *S. aureus* treated with protocatechuic acid (4× MIC; Figure 4a). There was similar increase in the fragmented DNA of protocatechuic acid (4× MIC)‐treated organisms (Figure 4b).

### 3.4 Electron complex inhibition

**Figure 5:**
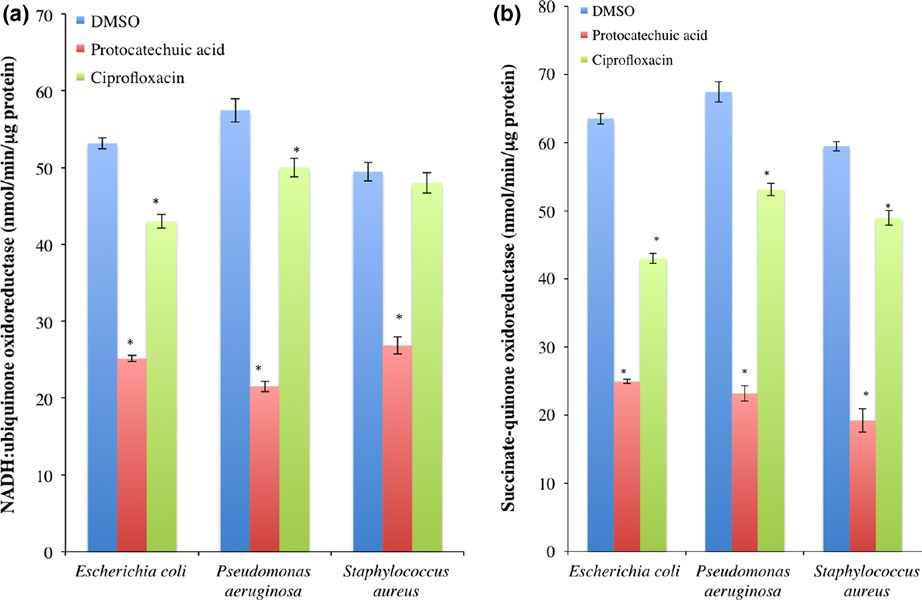
Activities of (a) NADH:ubiquinone oxidoreductase and (b) succinate:quinone oxidoreductase in *Escherichia coli* (ATCC 25922), *Pseudomonas aeruginosa* (ATCC 27853), and *Staphylococcus aureus* (ATCC 29213) exposed to protocatechuic acid (4× MIC). Values are mean ± SEM of three determinations and are statistically significant at *p *<* *.05. *p <* *.05 vs DMSO. DMSO, dimethyl sulfoxide

The activity of electron transport complex I in protocatechuic acid‐treated *E. coli*,* P. aeruginosa*, and *S. aureus* decreased significantly (*p *<* *.05) when compared with DMSO‐treated cells (Figure 5a). Similarly, protocatechuic acid decreased the activity of complex II in *E. coli*,* P. aeruginosa*, and *S. aureus* when compared to DMSO‐treated cells (Figure 5b). Although ciprofloxacin produced a decrease in the activities of electron transport complexes I and II in *E. coli*,* P. aeruginosa*, and *S. aureus*, it did not compared with protocatechuic acid.

### 3.5 Involvement of reactive oxygen species

**Figure 6:**
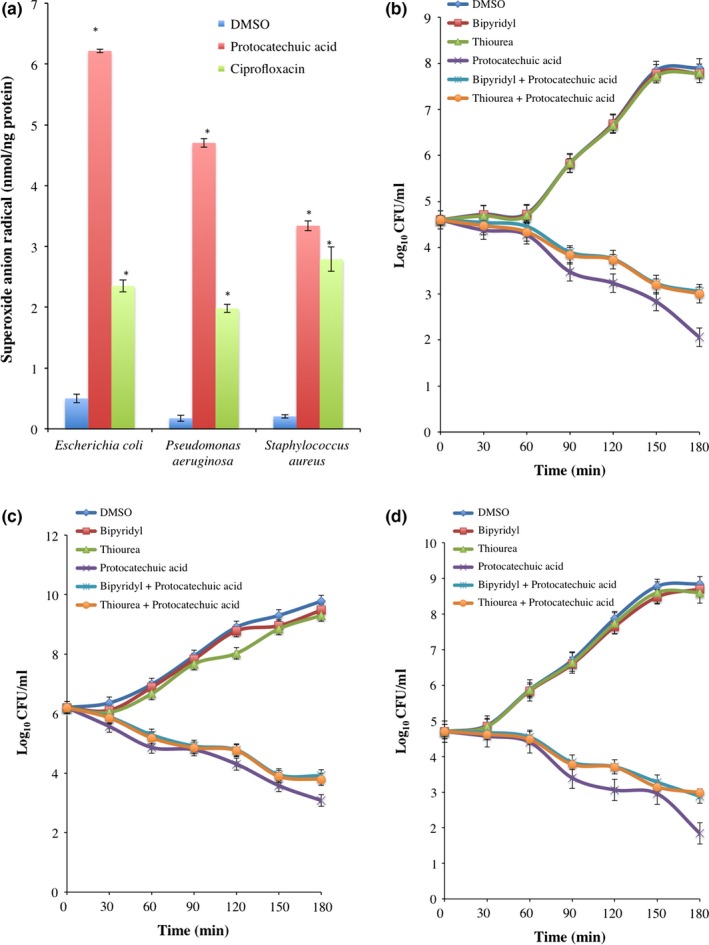
Involvement of reactive oxygen species in protocatechuic acid‐mediated bacterial death (a) superoxide anion radical content and viability (CFU/ml) of (b) *Escherichia coli* (ATCC 25922), (c) *Pseudomonas aureginosa* (ATCC 27853), and (d) *Staphylococcus aureus* (ATCC 29213) treated with protocatechuic acid (4× MIC) in the presence of 2,2′‐bipyridyl (500 μmol/L) and thiourea (150 mmol/L). Values are mean ± SEM of three determinations and are statistically significant at *p *<* *.05

The involvement of reactive species in protocatechuic acid‐mediated bacterial lethality by checking the superoxide content and 2,2′‐bipyridyl inhibition of hydroxyl radical generation via fenton chemistry. Superoxide anion contents of *E. coli*,* P. aeruginosa*, and *S. aureus* increased significantly (*p *<* *.05) following protocatechuic acid (4× MIC) treatment when compared to the DMSO‐treated cells (Figure 6a). It produced 12.44‐, 27.34‐, and 16.29‐fold increase in superoxide contents of *E. coli*,* P. aeruginosa*, and *S. aureus*, respectively. Although ciprofloxacin produced similar increase in superoxide anion radical, it did not compared with protocatechuic acid.

There was significant increase in CFU/ml of *E. coli*,* P. aeruginosa*, and *S. aureus* treated with only 2,2′bipyridyl (500 μmol/L) and thiourea (150 mmol/L) when compared with protocatechuic acid‐treated organisms (Figure 6b–d). Although protocatechuic acid in combination with 2,2′bipyridyl or thiourea decreased the number of viable cells, it did not compare with organisms treated with only lophirone B and lophirone C (Figure 6b–d).

## 4 Discussion

The golden mean to healthy living is maintaining redox homeostasis, as any imbalance could result to the production of oxidized cellular macromolecules. In this study, we present the involvement of oxidative stress in protocatechuic acid‐mediated bacterial lethality.

Protocatechuic acid has been demonstrated to be potent antimicrobial agents with wide spectrum activity (Da‐Costa‐Rocha et al., 2014; Liu, Tsao, & Yin, 2005). The MIC and MBC values generated in this study are in consonance with previous studies, indicating that bacteriostatic and bactericidal activities of protocatechuic acid are dependent on the concentration. These values are supported by decrease in absorbance and CFU/ml for 3 hr incubation. Similar antibacterial activity of this compound was reported with the pure form and derivatives (Liu et al., 2005).

Imbalance in the antioxidant defense system and ROS generation leading to oxidative damage of cellular macromolecules such as proteins, lipids, and DNA have been implicated in antibiotics and antimicrobials mediated bacterial lethality (Ajiboye et al., 2016a,b; Dwyer et al., 2014; Lobritz et al., 2015; Samoilova, Smirnova, Muzyka, & Oktyabrsky, 2014; Wang, Zhao, Malik, & Drlica, 2010; Zhao & Drlica, 2014; Zhao, Hong, & Drlica, 2015). Generation of ROS (superoxide anion radical) have been demonstrated in the prooxidant activity of protocatechuic acid (Zeraik et al., 2014). The bacteria (*E. coli*,* P. aeruginosa*, and *S. aureus*) responded to this increase by enhancing the activity of SOD and CAT. This increase could be an attempt to detoxify elevated ROS (H_2_O_2_ and •O_2_^−^).

Bacterial GSH complements cellular antioxidants by detoxifying H_2_O_2_ (Smirnova, Muzyka, & Oktyabrsky, 2012). Protocatechuic acid‐mediated depletion of GSH level in *E. coli*,* P. aeruginosa*, and *S. aureus* could perturb redox status and result to death. Although in a different cell culture, Babich, Sedletcaia, Kenigsberg, Babich, and Al (2002) reported similar GSH depletion by protocatechuic acid which is in consonance with our findings. Concomitant elevation in glutathione disulfide in protocatechuic acid‐treated bacteria evidently suggested the oxidation of GSH to GSSG. Consistent with our findings, esterified protocatechuic acid also led to the oxidation of GSH (Zeraik et al., 2014). The depletion of GSH and increased GSSG generation could distort redox balance resulting to oxidative attack on cellular macromolecules. Bactericidal agents have been reported to stimulate energy generating pathways such as citric acid cycle and electron transport leading to influx of electron into the electron transport chain through the reducing equivalent, NADH (Kohanski et al., 2007; Kohanski, Dwyer, & Collins, 2010). Indeed, protocatechuic enhanced influx of electron via NADH as evident from the increased NAD^+^/NADH ratio, which could result to electron and superoxide anion production.

Cellular macromolecules, DNA, and lipids are primary target of ROS leading to fragmentation of DNA and peroxidation of lipids (Ajiboye, 2010; Ajiboye et al., 2010). As such, products of lipid peroxidation and DNA fragmentation are useful indicator of oxidative status in bacteria. The increased level of MDA and fragmented DNA of protocatechuic acid‐treated bacteria is consistent with previous result obtained in human leukemia cells (Tseng et al., 2000) and cultured human cells from oral tissue (Babich et al., 2002). This increase indicates oxidative modification of cellular macromolecules in *E. coli*,* P. aeruginosa*, and *S. aureus*, possibly resulting from enhanced ROS generation.

Complexes I and III are large responsible for superoxide anion radical generation (Lanciano et al., 2013; Markevich & Hoek, 2015). Superoxide anion radical generated are dismutated to H_2_O_2_, which undergoes Fenton reaction (in the presence of Fe^2+^) to produce hydroxyl radical. Although the increased NAD^+^/NADH ratio evidently shows increased electron transport and correlate with superoxide anion radical generated. The inhibition of complexes I and II are indication that complex III and other sources may be responsible for the increased superoxide anion generated in this study.

Reactive oxygen species, in particular •O_2_^−^ and •OH^−^, have been implicated and documented as common mechanism for antimicrobials and antibiotics (Ajiboye and Haliru, 2016; Ajiboye et al., 2016b; Foti, Devadoss, Winkler, Collins, & Walker, 2012; Kohanski et al., 2007; Wang & Zhao, 2009).The elevated level of •O_2_^−^ in this study indicates enhanced ROS generation, which could have resulted via autoxidation of protocatechuic acid and inhibition of electron transport chain complexes. To further show the importance of reactive oxygen species generation in protocatechuic acid‐mediated bacterial lethality, *E. coli*,* P. aeruginosa*, and *S*,* aureus* were treated with protocatechuic acid and or 2,2′ dipyridyl (Fe chelator) and thiourea (hydroxyl radical) scavenger. Although the direct target of hydrogen peroxide is discerned, it undergoes Fenton reaction in the presence of Fe^2+^ to generate hydroxyl radical. Increase in CFU/ml of *E. coli*,* P. aeruginosa*, and *S. aureus* following incubation with protocatechuic acid in the presence of 2,2′ dipyridyl or thiourea indicates the involvement of hydroxyl radical in bacterial lethality. This supports previous studies that have established the involvement of hydroxyl radical as mechanism of antibacterial agents (Ajiboye et al., 2016b; Kohanski et al., 2007).

## 5 Conclusion

**Figure 7:**
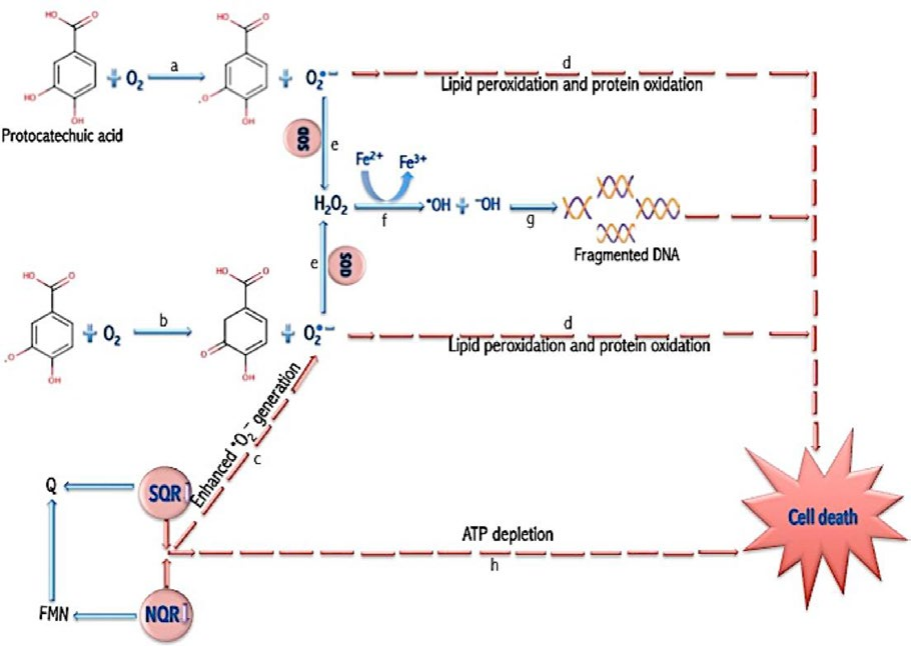
Proposed mechanism of protocatechuic acid‐mediated redox perturbation. (a) Autoxidation of protocatechuic acid generates •O_2_^**−**^ and semiquinone. (b) Semiquinone generated is further oxidized to generate more •O_2_^**−**^. (c) The inhibition of NQR and SQR further enhanced •O_2_^**−**^ generation. (d) •O_2_^**−**^ attacks polyunsaturated fatty acid components of membrane (lipid peroxidation) and thiol group of protein (protein oxidation). (e) Superoxide dismutase also converts •O_2_^**−**^ to H_2_O_2_. (f) In the presence of Fe^2+^, hydrogen peroxide undergoes Fenton reaction leading to the generation of •OH. (g) •OH attacks DNA bases resulting to DNA fragmentation. (h) Inhibition of NQR and SQR also results in ATP depletion. These events, lipid peroxidation, protein oxidation, DNA fragmentation and ATP depletion, result to cell death. NQR, NADH:quinone oxidoreductase; SQR, succinate:quinone oxidoreductase; MDA, malondialdehyde; GSSG, glutathione disulfide; SOD, superoxide dismutase; CAT, catalase; GSH‐Px, glutathione peroxidase; GSH, reduced glutathione

It is evident from the enhanced ROS generation, increased MDA and fragmented DNA, depleted reduced glutathione, and decreased respiratory chain activity that protocatechuic acid induced oxidative stress in its bacterial lethality against *E. coli*,* P. aeruginosa*, and *S. aureus* (Figure 7).

## Conflict of Interest

The authors declared no potential conflicts of interest with respect to the research, authorship, and/or publication of this article.
